# Incorporation of Extranodal Metastasis of Gastric Carcinoma into the 7th Edition UICC TNM Staging System

**DOI:** 10.1371/journal.pone.0019557

**Published:** 2011-06-13

**Authors:** Wei Wang, Yuanfang Li, Yu Zhang, Xiuhong Yuan, Dazhi Xu, Yuanxiang Guan, Xingyu Feng, Yingbo Chen, Xiaowei Sun, Wei Li, Youqing Zhan, Zhiwei Zhou

**Affiliations:** 1 State Key Laboratory of Oncology in South China, Sun Yat-sen University Cancer Center, Guangzhou, Guangdong, People's Republic of China; 2 Department of Gastric and Pancreatic Surgery, Sun Yat-sen University Cancer Center, Guangzhou, Guangdong, People's Republic of China; 3 Department of Pathology, Sun Yat-sen University Cancer Center, Guangzhou, Guangdong, People's Republic of China; The University of Hong Kong, Hong Kong

## Abstract

**Background:**

To assess the clinical significance and prognostic impact of extranodal metastasis (EM) in gastric carcinoma and establish an optimal classification in the staging system.

**Methodology/Principal Findings:**

A total of 1343 patients with gastric carcinoma who underwent surgical resection were recruited to determine the frequency and prognostic significance of EMs. EMs were divided into two groups (EM1 and EM2) and then incorporated into the 7^th^ edition UICC TNM staging system. EMs was detected in 179 (13.3%) of 1343 patients who underwent radical resection. Multivariate analysis identified EMs as an independent prognostic factor (HR = 1.412, 95%CI = 1.151–1.731, P<0.001). After curative operation, the overall survival rate were worse in patients with ≥3 cases of EM (EM2) than those with the number of 1 and 2 cases (EM1) (P<0.001). Survival of patients with EM1 was found almost comparable to that of N3 stage (P = 0.437). Survival of patients with EM2 showed similar to that of stage IV patients (P = 0.896). By using the linear trend X^2^, likelihood ratio X^2^, and Akaike information criterion (AIC) test, EM1 treated as N3 stage and EM2 treated as M1 stage performed higher linear trend X^2^ scores, likelihood ratio X^2^ scores, and lower AIC value than the 7^th^ edition UICC TNM staging system, which represented the optimum prognostic stratification, together with better homogeneity, discriminatory ability, and monotonicity of gradients.

**Conclusions/Significance:**

EMs might be classified based on their number and prognostic information and should incorporate into the TNM staging system.

## Introduction

Histological examination of dissected nodal structures may disclose the presence of nodules of tumors that are not contained with recognizable lymph node tissue. This kind of cancer deposit so called Extranodal Metastases (EMs), which comprising cancer cells in soft tissue discontinuous with the primary lesion without evidence of residual lymph node tissue, is found during routine examination of about 10–28 percent of resected gastric carcinoma specimens. The presence of EM has also been identified as a prognostic factor [1], [2].

It has long been ambiguous whether such involvement should be treated as a T, N, or even M factor, or should be excluded from consideration in determining tumor stage in recent years. In the American Joint Committee on Cancer (AJCC)/International Union Against Cancer (UICC) 5th and 6th edition TNM staging system, this type of tumor spread should be regarded as lymph node metastasis of the nodule with the form and smooth contour of a lymph node, but should otherwise be regarded as part of the primary tumor [3], [4]. In the year of 2009, UICC published the 7th edition TNM classification of malignant tumors for gastric carcinoma. According to the 7th edition classification, this type of metastatic nodules in the fat adjacent to a gastric carcinoma, without evidence of residual lymph node tissue, are considered regional lymph node metastases, but nodules implanted on peritoneal surfaces are considered distant metastases [5]. While this type of definition is still comprehensive and ambiguous. For example, should a case of EMs be deemed as a metastatic lymph node, or should a case of EMs be deemed as an upgraded N stage? With different features of EMs such as number or shape, the prognostic significance maybe still variable. Furthermore, how to combine this type of metastatic nodules into TNM staging system is also unclear. Up to date, there has been few studies focused on the significance through convincing analysis in gastric carcinoma [2], and there are even less data on the optimal categorization of such foci. Nevertheless, an optimal categorization should heighten the value of the TNM classification as a prognostic staging system.

Therefore, the aim of the present study was to assess the incidence, the relationship with other clinicopathologic factors, and the prognostic significance of EMs in patients with gastric carcinoma. In addition, to investigate the possible classification of EMs, we classified them into several different categories based on the different survival outcomes and determined whether EMs should be combined into TNM staging system or into which kind of staging. Finally, validation and comparison of the homogeneity, discriminatory ability and monotonicity of gradient of the new classification with the 7th edition TNM staging system were performed.

## Methods

### Participants

Clinicopathological data from 1580 cases of gastric cancer patients who underwent surgical resection from Jan. 1994 to Dec. 2006 at Sun Yat-sen University Cancer Center were analyzed retrospectively. The routine postoperative pathological results included tumor size, histological type, margin, adjacent tissues and neighboring organs, lymphatic/venous invasion, retrieved lymph nodes, metastatic lymph nodes, and pTNM staging. The eligibility criteria included histologically confirmed R0 resection, which was defined as no macroscopic and microscopic residual tumor and a postoperative survival time of ≥3 months. Patients with distant metastases and carcinoma of the gastric stump after gastric resection for benign disease were excluded from the study. Among the potential participants, 106 had distant metastases (liver, lung, ovary, abdominal or pelvic cavity dissemination), 41 underwent R1 or R2 resection, 13 had distant lymph node metastases (retropancreatic, mesenteric, duodenohepatic ligament or para-aortic lymph node), 15 died less than 3 months after resection, and 62 were lost to follow-up. Thus, 237 patients were excluded and 1343 patients were recruited.

### Surgical procedures

D2 lymphadenectomy was performed by experienced surgeons following the JGCA guidelines [6]. All resected specimens were fixed in 10 percent formalin, embedded in paraffin, and stained with haematoxylin and eosin. All solid structures in adipose connective tissue resected with the stomach were retrieved, including the lymph nodes and any areas of EMs. EMs were defined as the presence of cancer cells in soft tissue that was discontinuous with the primary lesion or in peri-stomach soft tissue distinct from the lymph node.

### Ethics statement

The protocol was approved by Sun Yat-sen University Cancer Center review board in keeping with Chinese bioethical regulations. All patients gave a written informed consent before participating in the study.

### Follow up

Postoperative follow-up included clinical and laboratory examinations every 3 months for the first 2 years at our outpatient department, every 6 months from the third to fifth years, and annually thereafter until at least 5 years after the operation or until the patient died, whichever came first. Overall patient survival, defined as the time from operation to death or last follow-up, was used as a measure of prognosis. The follow-up was closed in May 2010. The median follow-up for the entire cohort was 50 months (range 3–197 months).

### Statistical Analysis

Mann–Whitney U test and X^2^ tests were used where appropriate to compare the distribution of individual variables between groups. The 5-year survival rate was calculated using the Kaplan–Meier method. Statistical comparisons of different factors were made with the log-rank test. In multivariate analysis, forward stepwise regression analysis was performed with a Cox proportional hazards model. A two-tailed P value of ≤0.05 was considered statistically significant.

To compare the redefinitions of the T, N, and M categories with the 7th edition TNM staging system, the likelihood ratio X^2^ test related to the Cox regression model was used for measuring homogeneity. The discriminatory ability and monotonicity of gradient assessments were measured with the linear trend X^2^ test [6], [7], [8]. The Akaike information criteria (AIC) value within a Cox proportional hazard regression model also was calculated for each category to measure its discrimination ability and identify the optimal categorization of EMs that afforded the T, N, and M stages the highest power of discrimination of survival outcome for each stage [9]. The AIC (AIC = −2×Log likelihood +2× No. of parameters in the model) is an estimate of the measure of fit of a model to a given set of data. The model of choice achieves parsimony with maximum likelihood and the lowest value of AIC, indicating the smallest loss of information for predicting outcome [10], [11], [12], [13]. All statistical analyses were performed using SPSS software version 18.0 (SPSS Inc., Chicago, IL).

## Results

Of the 1343 patients, 902 (67.2%) were males, and 441 (32.8%) were females. The mean age of the patients was 56.4±11.9 years (range 17–85 years). The overall 5-year survival rate for all patients was 55.9%, and 730 patients were alive when our follow-up was completed.

Stage distribution included 192 (14.3%) patients with stage I, 408 (30.4%) patients with stage II, 743 (55.3%) with stage III according to the 7^th^ edition TNM staging system [5]. Consideration of the possible origin for EMs excluded from implantation on peritoneal surfaces, we investigated the incidence of EMs for 1343 cases of patients with potential radical resection.

**Figure 1:**
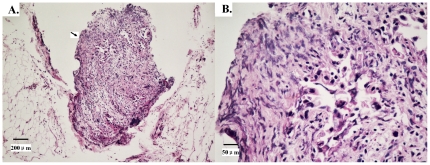
Haematoxylin and eosin staining shows extranodal metastasis (EM) in gastric carcinoma. Tumor cells are scattered into the resected adipose connective tissue around the stomach distinct from the metastatic lymph node. **1A**: Original magnification ×100, arrow indicates the EM. **1B**: Original magnification×400.

Overall, EMs were detected in 179 (13.3%) of the 1343 patients and in 359 (1.8%) of the 20,047 nodules retrieved as ‘lymph nodes’. In the 179 patients with EM, 102 patients were detected with 1 EM, 29 patients with 2 EMs, and 48 patients with ≥3 EMs. The mean number of metastases of this type was 1.44 (median 1.37, range 1–7). Figure 1 shows an example of EMs.

The incidence of EMs was significantly higher in patients with total gastric carcinoma, larger tumors (>5.0 cm) and in those with poorly and undifferentiated carcinoma (G3/G4). EMs was found significantly more common in tumors with lymphatic/venous invasion. Additionally, patients with EMs had a significantly deeper tumor invasion (T4a and T4b) and more number of lymph node metastases (N3). Tumor stage was III in 141 (78.8%) of 179 patients with EMs. (Table 1)

**Table 1:** Correlation between Extranodal Metastasis and clinicopathological factors in gastric carcinoma patients with potential radical resection.

Variable	Extranodal Metastasis		P value
	Positive (%)N = 179	Negative (%)N = 1164	
Gender			0.426
Male	125 (13.9)	777 (86.1)	
Female	54 (12.2)	387 (87.8)	
Age (year)			0.674
<60	97 (12.8)	662 (87.2)	
≥60	82 (14.0)	502 (86.0)	
Tumor location			<0.001
Proximal	96 (13.3)	628 (86.7)	
Distal	62 (10.9)	505 (89.1)	
Total	21 (40.4)	31 (59.6)	
Tumor size (cm)			<0.001
≤5.0	78 (9.8)	714 (90.2)	
>5.0	101 (18.3)	450 (81.7)	
Histological grade			<0.001
Well/Moderately differentiated (G1+G2)	38 (7.6)	465 (92.4)	
Poorly differentiated (G3)	97 (16.9)	476 (83.1)	
Undifferentiated (G4)	44 (16.5)	223 (83.5)	
Lymphatic/Venous invasion			<0.001
No	146 (11.8)	1096 (88.2)	
Yes	33 (32.7)	68 (67.3)	
Depth of invasion (AJCC 7th edition)			<0.001
T1	0 (0)	118 (100.0)	
T2	4 (2.3)	167 (97.7)	
T3	31 (12.2)	223 (87.8)	
T4a	120 (17.8)	555 (82.2)	
T4b	24 (19.2)	101 (80.8)	
Nodal status (AJCC 7th edition)			<0.001
N0	24 (5.2)	440 (94.8)	
N1	37 (13.9)	230 (86.1)	
N2	43 (13.1)	285 (86.9)	
N3	75 (26.4)	209 (73.6)	
TNM staging (AJCC 7th edition)			<0.001
Stage I	2 (1.0)	190 (99.0)	
Stage II	36 (8.8)	372 (91.2)	
Stage III	141 (19.0)	602 (81.0)	

Regarding survival, univariate analysis was performed in 1343 patients who underwent potentially radical resection. Variables including age, tumor location, size, differentiation, lymphatic/venous invasion, EMs, pT, pN, and TNM staging correlated well with the patients' life expectation. In the multivariate analysis, EMs emerged as an independent prognostic factor for survival together with the aforementioned factors (HR = 1.412, 95%CI = 1.151–1.731, P<0.001). (Table 2)

**Table 2:** Univariate and multivariate survival analysis of clinic-pathologic variables in 1343 cases of gastric carcinoma patients with potential radical resection.

Variables	Univariate analysis			Multivariate analysis		
	HR	95%CI	P value	HR	95%CI	P value
Gender (female vs. male)	0.962	0.814–1.136	0.645			
Age (year), (≥60 vs. <60)	1.433	1.226–1.675	<0.001	1.468	1.254–1.719	<0.001
Location (distal/proximal/total)	0.749	0.646–0.868	<0.001	0.720	0.625–0.829	<0.001
Size (cm) (>5 vs. ≤5)	2.110	1.804–2.468	<0.001	1.601	1.365–1.878	<0.001
Differentiation (G3/G2/G1)	1.257	1.133–1.393	<0.001	1.184	1.061–1.321	0.003
Lymphatic/Venous invasion (Yes vs. No)	2.038	1.408–2.832	<0.001	1.814	1.319–2.445	<0.001
EM (Positive vs. Negative)	2.362	1.949–2.862	<0.001	1.412	1.151–1.731	<0.001
T (T4b/T4a/T3/T2/T1)	1.811	1.654–1.984	<0.001	1.478	1.338–1.632	<0.001
N (N3/N2/N1/N0)	1.650	1.538–1.769	<0.001	1.417	1.311–1.531	<0.001

**Figure 2:**
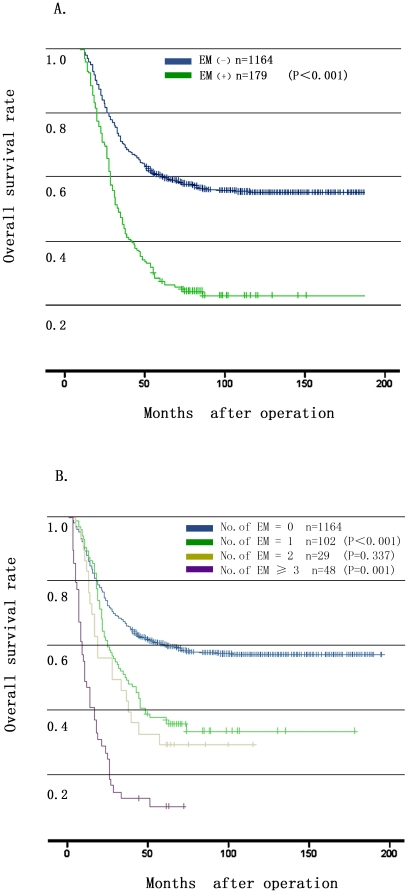
Prognostic significance of extranodal metastasis (EM) on overall survival rate of the gastric carcinoma patients underwent curative surgery. **2A**: A positive EM was significantly associated with a shorter survival time (P<0.001). **2B**: Overall survival curves of gastric carcinoma patients stratified by EM number (0, 1, 2, ≥3), (P<0.001, P = 0.337, P = 0.001, respectively).

Positive EMs were significantly associated with a shorter survival time (P<0.001) (Fig. 2A). Analysis of patients grouped according to the number of EMs revealed that patients with EMs ≥3 had an even worse postoperative survival than that of patients with the number of EMs less than 3. The 5-year survival rate of patients with the number of EMs as 1, 2, and ≥3 were 35.2%, 27.6%, and 6.8% (P = 0.337 and 0.001, respectively) (Fig. 2B).

In order to incorporate the EMs into the T, N, and M categories, we divided EMs as two separate groups, EM1 (with the number of EMs <3) and EM2 (with the number of EMs ≥3), according to the comparable survival outcomes between patients with the number of 1 and 2 EMs and the distinct survival outcomes between patients with the number of 2 and ≥3 EMs.

**Figure 3:**
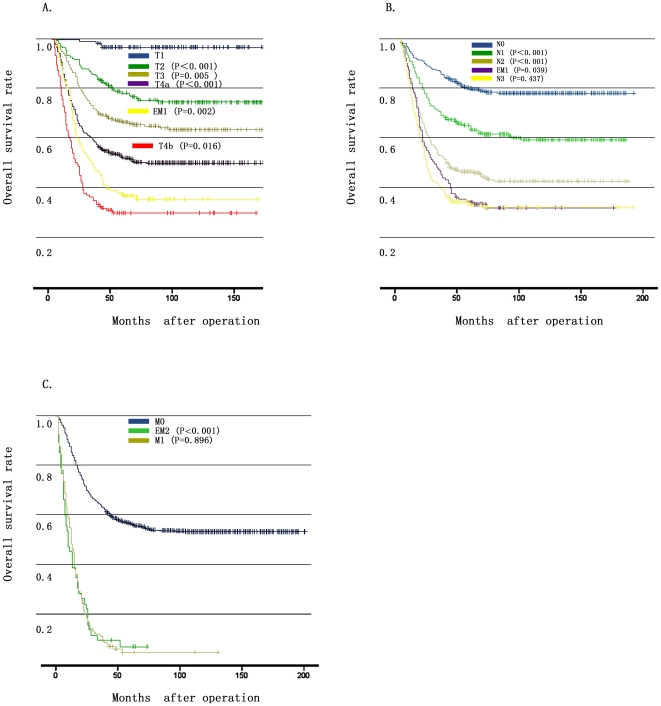
Comparison of survival curves between EMs and the T, N, M stages. **3A**: Overall survival curves showed different outcomes among patients with T4a, EM1 and T4b (P = 0.002 and 0.016, respectively). **3B**: Overall survival of patients with EM1 was worse than those of the N2 stage and was comparable to those of the N3 stage (P = 0.039 and 0.437, respectively). **3C**: Patients with EM2 had a comparable overall survival with those of the M1 stage (P = 0.896).

First, we compared the survival curve between patients with EM1 and T stage. The Kaplan-Meier plots showed a good discriminatory ability between patients with pT4a stage and patients with EM1 (5-year survival rate 48.8% vs. 32.6%, P = 0.002), also between patients with EM1 and those with T4b stage (5-year survival rate 32.6% vs. 23.4%, P = 0.016) (Fig. 3A). Secondly, when we compared the survival outcomes between patients with EM1 and N stage, the overall survival rate of patients with EM1 was found almost comparable to that of patients with N3 stage (5-year survival rate 31.3% vs. 32.6%, P = 0.437) and was found significant worse than that of patients with N2 stage (5-year survival rate 32.6% vs. 44.1%, P = 0.039) (Fig. 3B). Thirdly, when we investigated the possible origination of EM2 from peritoneal seeding, we found that the overall survival rate of patients with EM2 showed similar to that of aforementioned 106 cases of stage IV patients. The 5-year survival rate was 6.8% and 5.6%, respectively (P = 0.896) (Fig. 3C).

**Figure 4:**
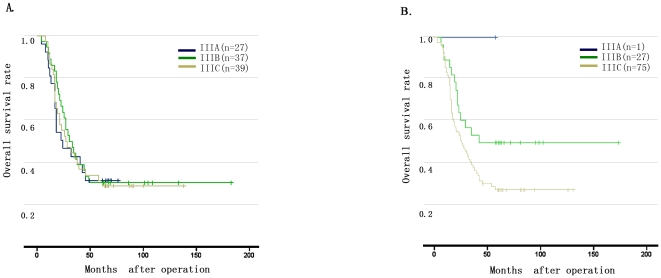
Comparison of survival curves among 103 cases of stage III patients with EM1 before and after consideration of EM1. **4A**: Patients with EM1 had a comparable overall survival curves among each substage when EM1 was ignored (P = 0.991). **4B**: Patients with EM1 had a distinguishable overall survival curves among each substage when with the consideration of EM1 as N3 stage (P = 0.023).

Furthermore, we realized that 78.8% (141 of 179) of the EMs was mainly found in stage III patients, with 103 cases of EM1 and 38 cases of EM2. In order to investigate the incidence and influence of EM1 in stage III patients, we analyzed the survival outcomes with and without consideration of EM1 as N3. Without consideration of EM1, the distribution of stage III was 27 cases of stage IIIA, 37 cases of stage IIIB, and 39 cases of stage IIIC patients. The survival curves were shown in Fig. 4A, in which the survival difference in each substage was confused (P = 0.991). When we treated the EM1 as N3 patients, the distribution of stage III was changed as 1 case of stage IIIA, 27 cases of stage IIIB, and 75 cases of stage IIIC patients. The survival curves were shown discriminatory in each substage (P = 0.023) (Fig. 4B).

In terms of the results aforementioned, different survival outcomes based on the T, N and M stage were compared among the following 4 redefinitions of the T, N and M categories: A, the 7th edition TNM definition without considering EMs; B, redefinition of T stage with EM1 treated as T_EM_ stage; C, redefinition N stage with EM1 treated as N3 stage; D, redefinition of M stage with EM2 treated as M1. The performance of the 7th edition TNM staging systems with redefinitions of the T, N, and M categories was assessed by the linear trend X^2^, likelihood ratio X^2^, and the AIC tests, which was described in Table 3. Compared with the 7th edition staging system without consideration of EMs, the redefinition of T stage treated with EM1 as T_EM_ stage, redefinition of N stage with EM1 treated as N3 stage and redefinition of M stage with EM2 treated as M1 stage all had higher linear trend X^2^ scores and likelihood ratio X^2^ scores, and lower AIC value, which represented the optimum prognostic stratification, together with better homogeneity, discriminatory ability, and monotonicity of gradients. (Table 3)

**Table 3:** Definitions of T, N and M categories and their impact on the prognostic value of staging.

Category	Definition	Subgroups	Linear Trend X^2^	Likelihood Ratio X^2^	*AIC
A	7th ed. T stage (n = 1343)	T1, T2, T3, T4a, T4b	171.755	201.840	8556.400
	7th ed. N stage (n = 1343)	N0, N1, N2, N3	184.258	194.901	8547.653
	7th ed. M stage (n = 1503)	M0, M1	112.316	133.065	10488.061
B	Number of 1 and 2 EMs (EM1) treated as T_EM_ stage (n = 1343)	T1, T2, T3, T4a, T_EM_,T4b	201.580	235.795	8515.847
C	Number of 1 and 2 EMs (EM1) treated as N3 stage (n = 1343)	N0, N1, N2, N3	235.104	247.322	8508.678
D	Number of ≥3 EMs (EM2) treated as M1 stage (n = 1503)	M0, M1	153.143	179.471	10421.502

*AIC = Akaike information criterion;

## Discussion

Dependent on the pathological examination, a large variation in the incidence of EMs has been reported in previous studies, ranging from 10 to 28% of cases [1], [2], mainly due to less caution about this special type of cancer deposit and its prognostic value. In the present study, we investigated the clinical parameters and the prognostic value of EMs in a group of patients who underwent potentially radical resection for gastric carcinoma, in which we found that the incidence of EMs was 179 (13.3%) in 1343 patients. The results revealed that the presence of EMs had a significant correlation with the total gastric carcinoma, larger tumor, poorly and undifferentiated carcinoma (G3/G4), lymphatic/venous invasion, deeper tumor invasion (T4a and T4b) and more number of lymph node metastases (N3), which were proven with worse survival outcomes. In multivariate analysis, present of EMs maintained as an independent prognostic factor for gastric carcinoma patients with poor postoperative survival (HR = 1.412, 95%CI = 1.151–1.731, P<0.001). This phenomenon is consistent with the finding that EMs showed a close correlation with cancer aggressiveness reported in previous studies. While in previous studies, the patients who were found peritoneal seeding in the operation were also enrolled and investigated [1], [2]. In terms of the 7th edition UICC staging system of gastric carcinoma, the type of EMs implanted on peritoneal surfaces are clearly deemed as distant metastases (stage IV). So our study mainly investigated the classification of EMs in patients underwent potential radical resection and excluded patients with obvious peritoneal seeding revealed during operation. Firstly, we found that the survival outcomes were similar between patients with the number of 1 and 2 cases of EMs and distinct with patients with ≥3 cases of EMs, which may indicate different origin and classification of this type of metastasis. Secondly, we divided EMs into two separate groups (EM1 and EM2) according to aforementioned distinct survival outcomes and investigated the possible classification. In T stage, we found that the 5-year survival rate was different among patients with T4a, EM1 and T4b. In N stage, the 5-year survival rate of patients with EM1 was comparable to those of N3 stage and was worse than those of N2 stage. In M stage, the result revealed that patients with EM2 had a comparable survival with those of M1stage. Furthermore, with the consideration of EM1 mainly in stage III patients, we investigated the influence of EM1 in each substage of stage III patients. The results also supported that EM1 should be considered as N3 stage, if not, the survival outcomes will be confused in each substage. Finally, we validated our results utilizing the linear trend X2, likelihood ratio X2, and the AIC tests to confirm the homogeneity, discriminatory ability, and monotonicity of gradients of our novel classification. Compared with the 7th edition TNM staging system without consideration of the EMs, the new categories for N3 and M1 stage demonstrated that they performed better homogeneity, discriminatory ability, and monotonicity of gradients.

Although little evidence is available on the prognostic value of EMs in gastric carcinoma patients, the presence of EMs was incorporated in the TNM staging manuals in 1997 for the first time, in which EMs were deemed as T or N stage in terms of their shape [3]. The shape of tumor deposits is, however, not sufficient to consistently distinguish different types of tumor involvement of the perivisceral fat [14]. In our opinions, classification of EMs based on their shapes is relatively subjective, insufficiently validated, and difficult to clinical utilizing. In colorectal cancer, some authors suggested that EMs should not be classified on the basis of the shape but according to their origin [14], [15]. Furthermore, in our present study, we demonstrated that the prognosis of patients with EM1 were distinct to neither T4a nor T4b patients, which indicated that the EMs should be not classified into T stage. In the 7th edition system, the classification of EMs was revised and updated to either to N or M stage although it was still relatively ambiguous [5]. Tanaka et al. revealed that the extranodal invasion was a significant risk factor for peritoneal metastasis in gastric carcinoma that EMs maybe indicated to M category [1]. Puppa et al. suggested that such tumor extension represented peritoneal seeding from either the primary tumor or metastatic lymph nodes and may rather be included in the M category for staging purposes as they represented in-transit metastases, both in colorectal cancer and other adenocarcinomas such as gastric, biliary duct and pancreatic carcinomas [14], [16]. For the aspect of clinical practice, one of the main objectives of the TNM classification is to provide prognostic information useful for deciding the best treatment options for the patients, stratifying them into groups that are prognostically and therapeutically similar [17]. In our study, we intended to incorporate the EMs into TNM staging system according to its prognostic information which differed with previous proposals. Although previous study has demonstrated that the number of EMs was correlated with poor prognosis of gastric carcinoma [2], in our study number of EMs, for the first time, was taken into account for classification of TNM staging. To validate the feasibility of our assignment for EM1 and EM2, methods of linear trend X2, likelihood ratio X2, and the AIC tests were used. According to Ueno et al. [18], the performance of the staging system can be evaluated as homogeneity within subgroups, discriminatory ability between different groups, and monotonicity of gradients shown in the correlation between stages and survival rates. In our study, the new categories of EM1 and EM2 incorporating into N3 and M1 staging performed better homogeneity (higher likelihood ratio X2 score), discriminatory ability, and monotonicity of gradients (higher linear trend X2 score). More importantly, the new categories also showed smaller AIC value, representing the optimum prognostic stratification and indicating the smallest loss of information for predicting outcome. These results demonstrated better prognostic stratifications of our assignment for EMs than the 7th edition TNM staging system without consideration of EMs.

We acknowledge several limitations in this study. Our sample population is from a single institution experience and relatively small compared with the worldwide gastric cancer collaboration database, and is based on a retrospective study. While the strengths of this study are that the surgical procedures, pathologic examinations, and patient follow-up were uniform throughout the entire study period. Cancer staging is a dynamic process. As our understanding of cancer biology improves, the TNM staging system will need to be revised accordingly. Our present study demonstrated the EMs as an important prognostic factor and for the first time intended to incorporate it into N3 or M1 staging according to its number retrieved in postoperative samples in patients with gastric carcinoma.
